# A case report of thyroid-like low-grade nasopharyngeal papillary adenocarcinoma with literature review

**DOI:** 10.3389/fonc.2026.1811898

**Published:** 2026-06-02

**Authors:** Dehui Kong, Man Yang, Xie Deng, ZiFan Xu, Hongqiong Duan, Guoqing Pan, Zi Lei

**Affiliations:** 1 Department of Pathology, First Affiliated Hospital of Kunming Medical University, Kunming, China; 2 Department of Pathology, Fuyuan People’s Hospital, Qujing, China; 3 Department of Pathology, Zhenxiong People’s Hospital, Zhaotong, China

**Keywords:** case report, literature review, nasopharyngeal carcinoma, pathological characteristics, thyroid-like low-grade nasopharyngeal papillary adenocarcinoma

## Abstract

**Objective:**

This paper aims to systematically investigate the clinical and pathological characteristics of TL-LGNPPA and its differential diagnosis, with the objective of enhancing our understanding of this tumor and facilitating the development of more precise treatment strategies.

**Methods:**

A systematic review and meta-analysis of the clinical and pathological features, as well as differential diagnosis, of a case of TL-LGNPPA were conducted, and a comprehensive literature review was conducted by analyzing relevant articles published on PubMed.

**Results:**

The histological features of TL-LGNPPA are typically characterized by typical papillary and ductal structures, with fibrovascular axis present in the interstitium. Focal nuclear grooves may occasionally be seen, with moderate degree of atypia. Immunohistochemical analysis showed that all tumors expressed TTF-1 and CK, but TG was mostly negative, and the Ki-67 proliferation index was generally less than 10% in most cases.

**Conclusions:**

TL-LGNPPA is a rare nasopharyngeal tumor with histological features similar to papillary thyroid carcinoma. An accurate diagnosis requires a comprehensive consideration of histological morphological assessment, immunohistochemical analysis and imaging examination.

## Introduction

Thyroid-like low-grade nasopharyngeal papillary adenocarcinoma (TL-LGNPPA) is a rare low-grade epithelial neoplasm first described and reported by Carrizo et al. in 2005 (1). Nasal obstruction is the most common clinical presentation of TL-LGNPPA, and patients may also experience nasal bleeding, rhinorrhea, and sputum with blood. However, they may also have no apparent symptoms and be incidentally discovered during endoscopic examination (2, 5). The tumor typically appears grayish red in color and is soft in texture, growing slowly with a maximum diameter range of 0.4 to 4 cm, displaying indolent biological behavior and good prognosis. TL-LGNPPA typically exhibits typical papillary and glandular structures in histology, with a fibrous vascular axis within the stroma and focal nuclear grooves. Its degree of atypia is mild to moderate, while nuclear grooves are rarely seen (3). Immunohistochemical studies show that all TL-LGNPPA express thyroid transcription factor-1 (TTF-1) and cytokeratin (CK), while the Ki-67 index is usually ≤10% (4).

TL-LGNPPA is a rare tumor that has only been reported in a few cases both domestically and internationally, so most clinicians and pathologists have limited knowledge of it. This study aims to enhance our understanding of TL-LGNPPA by analyzing its clinical features, histopathological manifestations, and differential diagnosis, thereby improving the accuracy of pathological diagnosis.

## Materials and methods

### Cases

A TL-LGNPPA consultation case from the First Affiliated Hospital of Kunming Medical University meets the diagnostic criteria for TL-LGNPPA as stipulated in the 2022 WHO Classification of Head and Neck Tumors.

By conducting PubMed literature search (http://www.ncbi.nlm.nih.gov/pubmed/) using multiple keyword combinations related to the title and abstract, such as “thyroid-like low-grade nasopharyngeal papillary adenocarcinoma”, “low-grade nasopharyngeal papillary adenocarcinoma”, “thyroid-like nasopharyngeal papillary adenocarcinoma”, and “nasopharyngeal papillary adenocarcinoma”, we reviewed the relevant published literature and extracted the necessary clinical pathological data. The initial database search effectively identified a total of 123 articles potentially relevant to our research topic. Subsequently, only cases confirmed as TL-LGNPPA were retained. Through a meticulous selection process, we finally incorporated 30 articles that most closely aligned with the scope and objectives of our study. These selected articles together constituted a comprehensive dataset, encompassing a total of 58 individual cases for further analysis and discussion. Detailed clinical data from this case as well as previously reported cases are summarized in **Table 1**.

**Table 1:** Summary of clinical data in the TL-LGNPPA literature.

Author	Gender	Age	Size (cm)	Location	IHC (-)	IHC (+)	Treatment	Recurrence or metastasis
Chen W et al. (2)	M	44	1.1	posterior wall of the nasopharyngeal roof	TG, PAX8, P63, P40, S100, P16, SOX10, Napsin A	CK, EMA, CK7, CK19, TTF-1, EBER, Ki-67 (5%)	Surgery	NO
Wang T et al. (3)	M	53	0.8	nasopharynx	MC, TG, PR, AR, CK5/6, PAX8, CDX2, CK20, NapsinA, Calponin1, WT1, GCDFP15, CA9, CD15, GATA3	TTF-1, CK19, EMA, CK7, ER80%3 + , Ki-67 (2%), CK5/6 focus, P63 focus, P53 a few cells	Surgery	NO
	F	39	2.2	posterior foramen of the nose	CK5/6, TG, NapsinA, PAX8, WT1, CA9, GCDFP15, CDX2, GATA3, PR, AR, CK20, Calponin1, P63, CK5/6, MC, CD15	CK7, TTF-1, ER focus, CK19, Ki-67 (5%), EMA, P53 a few cells	Surgery	NO
	F	43	0.7	Posterior wall of nasopharyngeal parietal	CDX2, TG, CA9, Calponin1, P63, PAX, WT1, GATA3, CK20, NapsinA, MC, CK20, GCDFP15, CD15, CK5/6, AR	TTF-1, CK7, ER80%3 +, CK19, PR60%2+, Ki-67 (3%), EMA, P53 a few cells	Surgery	NO
	F	31	0.7	nasopharynx	CK5/6, CK20, Calponin1, p63, CDX2, PAX8, GATA3, MC, NapsinA, GCDFP15, CA9, CD15, TG, AR, WT1, PR, GATA3	TTF-1, CK19, EMA, CK7, ER80%3 + , Ki-67 (2%), P53 a few cells	Surgery	NO
Wang L et al. (9)	F	15	3.0	Roof of the nasopharynx	TG, S-100, PAX-8, EBER	CK, CK7, CK19, TTF-1 focal , Ki-67 (1%)	Surgery	NO
	M	45	1	Roof of the nasopharynx	TG, S-100, PAX-8, EBER	CK, CK7, CK19, TTF-1, Ki-67 (3%)	Surgery	NO
	M	46	1.5	Depth of the nasal cavity	TG, S-100, PAX-8, EBER	CK, CK7, CK19, TTF-1 focal , Ki-67 (2%)	Surgery	NO
	F	67	1.3	Posterior end of the nasal septum	TG, S-100, PAX-8, EBER	CK, CK7, CK19, TTF-1, Ki-67 (1%)	Surgery	NO
Guo H et al. (4)	F	47	1.0	posterior edge of the nasal septum	CK5/6, PAX-8, S-100, SOX-10, GATA-3, BRAF-V600E, TPO, TG	CK19, CK7, TTF-1, Galectin-3, HBME-1, Ki-67 (1%)	Surgery	NO
Li L et al. (5)	F	35	0.8	posterior edge of the nasal septum	TG, CK20, S-100, P63, P40, SMA, CDX-2, GFAP	CK, CK7, CK19, VIM, TTF-1	Surgery	NO
Chen F et al. (6)	F	45		upper nasopharynx	CK20, TG	CK, CK19, CK7, CK8, EMA, TTF-1	Surgery+ Radiotherapy	NO
Takakura H et al. (7)	F	38	2.0	posterior edge of nasal septum	CK5/6, CK20, SMA, Calponin, P63, GFAP, S-100, CDX2, CEA, PAX8, CD10, DOG1, GATA3, SOX10	TTF1, CK19, VIM, EMA, CK7, Ki-67 (5%)	Surgery	NO
Lee SH et al. (8)	F	50	NA	roof of the nasopharynx.	PAX8, TG, P63, SMA, S100, CK5/6	TTF1, VIM, MC, CK7, CK19	Surgery	NO
Maocai L et al. (10)	F	50	1.2	posterior end of the nasal septum	TG, CK5/6, CD117	CK, VIM, TTF-1, CK19, Ki-67 (1%)	Surgery	NO
Baumann KB et al. (11)	M	26	NA	Junction of the posterior nasal cavity and nasopharynx	TG, CK5/6	TTF-1, CK7, EMA	Surgery	NO
Oide T et al. (12)	M	68	0.8	roof of the nasopharynx	PAX8, TG	TTF-1focal, CK5/6, P63, HCK	Surgery	NO
Ohe C et al. (13)	M	25	0.8	roof of the nasopharynx	TG, CD15, CK5/6, CK20, S-100	CK7, CK19, TTF-1, VIM	Surgery	NO
	F	41	0.5	posterior roof of the nasopharynx	TG, CD15, CK5/6, CK20, S-100	CK7, CK19, TTF-1, VIM	Surgery	NO
Huang F et al. (14)	F	26	1.5	Roof of the nasopharynx	PAX8, TG, CK5/6	TTF-1, CK19, S-100Focal, EMA, CK7, Ki-67 (5%)	Surgery	NO
	M	44	0.4	Roof of the nasopharynx	PAX8, TG, CK5/6, S-100	TTF-1, CK19, EMA, CK7, Ki-67 (2%)	Surgery+ Radiotherapy	NO
	M	19	1	Posterior superior edge of the nasal septum	PAX8, TG, CK5/6, S-100	TTF-1, CK19, EMA, CK7, Ki-67 (5%)	Surgery	NO
	M	29	1	Posterior edge of the nasal septum and the roof posterior wall of the nasopharynx	PAX8, TG, CK5/6	TTF-1, CK19, S-100Focal, EMA, CK7, Ki-67 (5%)	Surgery+ Radiotherapy	NO
	F	36	1.2	Roof of the nasopharynx	PAX8, TG, CK5/6	TTF-1, CK19, S-100Focal, EMA, CK7, Ki-67 (3%)	Surgery	NO
Zhang WL et al. (15)	M	64	2.0	posterior wall of the nasopharynx	TG, CK20, P63, P40, CK5/6, PSA, NapsinA, S100, GFAP, SMA, CDX2, PAX8	CK7, CK19, TTF-1, CEA	Surgery	NO
Oishi N et al. (16)	F	47	2.0	posterior edge of the left nasal septum	TG, TSG, PAX8, CK5/6, CK20, SMA, P63, S-100	VIM, CK7, CK19, MC, ER, Ki-67 ( 5%)	Surgery	NO
Le QV et al. (17)	F	50	4.0	nasopharynx	TG, CK5/6, CK20, SMA, P63, S-100	TTF-1, CK7, CK19	Surgery	NO
Wang J et al. (18)	F	42	0.5	junction of the nasopharyngeal wall and free margin of the nasal septum	/	TTF-1	Surgery	NO
Yi L et al. (19)	F	45	4.0	posterior nasal septum.	/	TTF-1, P40, P63, Ki-67 (3%)	Surgery	NO
Mirza R et al. (20)	M	54	1.0	Midline of the anterior-nasopharynx	CK5/6, S100, TG	CK19, TTF1, Ki-67< 2%	Surgery	NO
Horino T et al. (21)	F	25	1.7	Roof of the nasopharynx	TG, CK7, CK5/6, CK20, SMA, P63	TTF1, CK7, VIM, Ki-67 (5%)	Surgery	NO
Toyran T et al. (22)	M	70	0.3	left pharyngea	P53, TG, S100, actin, P16	TTF-1, EMA, CEA, CDX2, Ki-67 (60%)	Surgery	NO
Hu H et al. (23)	M	20	2.1	posterior wall of the nasopharynx	TG, S-100, GFAP	EMA, TTF1, CK, CK7, CEA Focal, CD117 Focal, Ki-67 (15%)	Surgery	NO
Borsetto D et al. (24)	F	15	NA	nasopharyngeal lesion originating from the bony ridge of the vomer	TG, CK20, P63, P40, CK5/6, PSA, NapsinA, S100, GFAP, SMA, CDX2, PAX8, P53, Bcatenin, TSH	CK, CK7, CK19, ER, Ki-67 (4%)	Surgery	NO
Li M et al. (25)	F	15	2.5	posterior nasal septum	CD15, S100, P40, CK20, CDX-2, GFAP	CK, CK7, TTF-1, VIM, EMA, Ki-67 (5%)	Surgery	NO
Sourati A et al. (26)	F	35	2.0	nasopharynx	TG, GFAP, CD57, CK5/6, P63, CDX2, CK20, CT, S-100	TTF-1, CK7, EMA	Surgery	NO
Carrizo F et al. (1)	M	9	2	right nasopharyngeal	CK5/6, CK20	CK7, CK19, TTF-1	Surgery	No
	M	13	1.5	junction of the nasal septum and the vault	CK5/6, CK20	CK7, CK19, TTF-1	Surgery	NO
Lai Y et al. (28)	F	56	NA	roof of the nasopharynx	TG	TTF-1, CK7, CK8, VIM, EMA, CK, Ki-67 (1%)	Surgery	NO
	F	15	NA	posterior margin of the nasal septum	TG	TTF-1, CK7, CK8, VIM, EMA, CK, Ki-67 (5%)	Surgery	NO
	M	40	NA	posterior margin of the nasal septum	TG	TTF-1, CK7, CK8, VIM, EMA, CK, Ki-67 (20%)	Surgery	NO
	F	30	NA	posterior margin of the nasal septum	TG	TTF-1, CK7, CK8, VIM, EMA, CK, Ki-67 (5%)	Surgery	NO
	F	25	NA	posterior margin of the nasal septum	TG	TTF-1, CK7, CK8, VIM, EMA, CK, Ki-67 (6%)	Surgery	NO
	M	31	NA	roof of the nasopharynx and posterior margin of the nasal septum	TG	TTF-1, CK7, CK8, VIM, EMA, CK, Ki-67 (15%)	Surgery	NO
	F	45	NA	roof of the nasopharynx	TG	TTF-1, CK7, CK8, VIM, EMA, CK, Ki-67 (1%)	Surgery	NO
	M	71	NA	roof of the nasopharynx	TG	TTF-1, CK7, CK8, VIM, EMA, CK, Ki-67 (1%)	Surgery	NO
	M	44	NA	roof of the nasopharynx and posterior margin of the nasal septum	TG	TTF-1, CK7, CK8, VIM, EMA, CK, Ki-67 (2%)	Surgery	NO
	M	26	NA	posterior margin of the nasal septum	TG	TTF-1, CK7, CK8, VIM, EMA, CK, Ki-67 (3%)	Surgery+ Radiotherapy	NO
	F	41	NA	roof of the nasopharynx	TG	TTF-1, CK7, CK8, VIM, EMA, CK, Ki-67 (5%)	Surgery	NO
	F	26	NA	posterior margin of the nasal septum	TG	TTF-1, CK7, CK8, VIM, EMA, CK, Ki-67 (2%)	Surgery	NO
	M	35	NA	roof of the nasopharynx	TG	TTF-1, CK7, CK8, VIM, EMA, CK, Ki-67 (5%)	Surgery	NO
	F	34	NA	roof of the nasopharynx	TG	TTF-1, CK7, CK8, VIM, EMA, CK, Ki-67 (3%)	Surgery	NO
Baba A et al. (29)	M	30	1.7	posterior margin of the nasal septum	NA	NA	Surgery	NO
	M	60	1.4	posterior margin of the nasal septum	NA	NA	Surgery	NO
	M	32	0.8	posterior margin of the nasal septum	NA	NA	Surgery	NO
Zhong QY et al. (27)	F	22	0.8	posterior roof of the nasopharynx	MC, CK5/6, CK20, P53, P63, S100, SMA, TG	VIM, CK19, TTF1, Glypican-3, CK7	Surgery	NO
Petersson F et al. (30)	F	39	1.0	posterior nasal septum	CEA, CT, CGA, S100, TG, CDX2, P63	CK, CK19, VIM, TTF-1, Ki-67 (3–4%)	Surgery	NO
Our study	F	28	0.8	roof of the nasopharynx	/	TTF-1, CK, TG, Ki-67 (5-10%)	Surgery	NO

### Paraffin pathological section examination

Organ samples were fixed in 10% neutral formaldehyde solution for 6–8 hours, followed by overnight dehydration. After tissue collection, the tissue was embedded in paraffin wax, and the wax block was sliced into 4 mm thick sections, which were heated in an oven for 30 minutes to ensure the effectiveness of routine dewaxing and benzene washing. After hematoxylin-eosin staining, the specimen was again washed, dehydrated, cleared, and mounted for observation under an optical microscope.

### Immunohistochemical staining

Immunohistochemical detection was performed using an optimized EnVision two-step method combined with diaminobenzidine (DAB) staining technique. The primary antibodies used were provided by Fuzhou MaiXin Biotechnology Co., Ltd. and selected primary antibodies included a broad spectrum of CK(MAB-0671), thyroglobulin(TG,MAB-0797), TTF-1(MAB-0677), and Ki-67 (MAB-0672).

## Results

### Case presentation

The case originated from a consultation at the First Affiliated Hospital of Kunming Medical University. The patient was a 28-year-old female who presented with nasal congestion lasting over 4 months. She also had mild discomfort in her nasopharynx, but no symptoms such as nosebleed, nasal discharge, dizziness, or headache. The computer tomography (CT) scan showed a nodular soft tissue protrusion on the dorsum of the nasopharynx, measuring approximately 14 mm×9 mm×10 mm. The lesion showed markedly heterogeneous enhancement during contrast-enhanced scanning, and the mucosa of the nasopharynx was slightly thickened, with marked enhancement during contrast-enhanced scanning. The parapharyngeal recesses were present on both sides and symmetrical. The floor of the fossa was not narrowed, and there was no destruction or resorption of the underlying bone in the skull base (**Figure 1**). Ultrasound showed normal thyroid gland, and thyroid function tests were normal. The patient underwent endoscopic total resection of the lesion.

**Figure 1:**
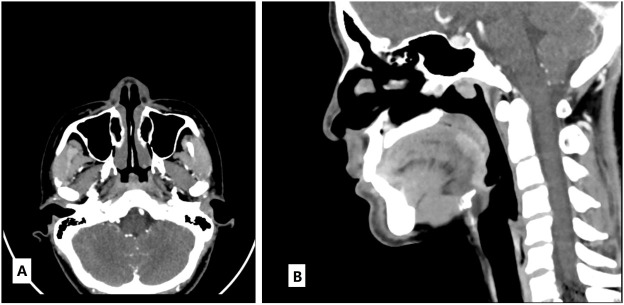
**(A)** a nodular soft tissue protrusion is observed at the roof of the nasopharynx. The bilateral pharyngeal recesses are present and symmetrical. The parapharyngeal space is not narrowed. No destruction or absorption of the skull base bone is noted (coronal view of enhanced CT). **(B)** A nodular soft tissue protrusion is present at the roof of the nasopharynx. The lesion shows significantly heterogeneous enhancement upon enhanced scanning. The mucosa of the nasopharynx is slightly thickened, and the mucosa is significantly enhanced upon enhanced scanning (sagittal view of enhanced CT).

### Pathological examination

Gross examination: A pedunculated polypoid mass of grayish-red color was observed, measuring approximately 0.8 cm × 0.6 cm × 0.4 cm, with a pedicle length of 0.2 cm. The section was grayish-red, solid, and soft in texture.

Microscopic manifestations: The tumor tissue exhibited a complex papillary architecture, within which fibrovascular axes were discernible, resembling thyroid papillary carcinoma. The papillary structures were intricate and closely arranged. The surface was covered by single-layer or pseudostratified columnar epithelium. Partial stroma presented with edema and sclerosis. The tumor cell nuclei were round or oval, with ground-glass-like appearance, the cytoplasm was clear or eosinophilic, the nucleoli were small, the nuclei were hyperchromatic, the tumor cells showed mild atypia, and mitotic figures were rare (**Figure 2**).

**Figure 2:**
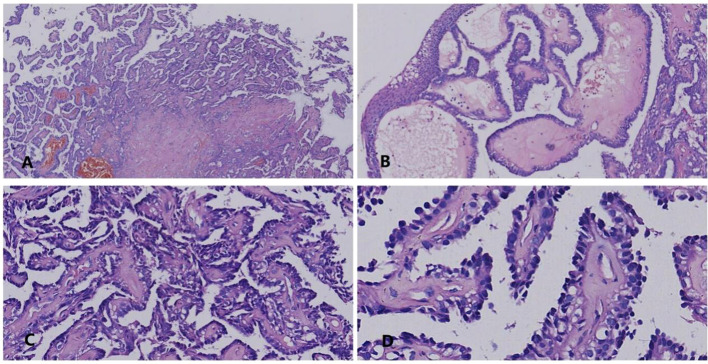
**(A)** presenting a complex papillary structure and a transparent fibrovascular axis (HE 40×); **(B)** the papillae are covered by single-layer or pseudostratified columnar epithelium, with interstitial edema and sclerosis (HE 100×); **(C)** the tumor cells are arranged in pseudostratified or single-layer columnar patterns, overlapping each other, covering the papillary areas. The cytoplasm is clear or eosinophilic, with small nucleoli and deeply stained nuclei (HE 200×); **(D)** the nuclei of the tumor cells are round or oval, with a ground-glass-like appearance (HE 400×).

Immunohistochemical staining results:TTF-1 and CK showed diffuse and strong positive expression, TG showed focal positive expression, and the Ki-67 index was relatively low, approximately 5-10% (**Figure 3**).

**Figure 3:**
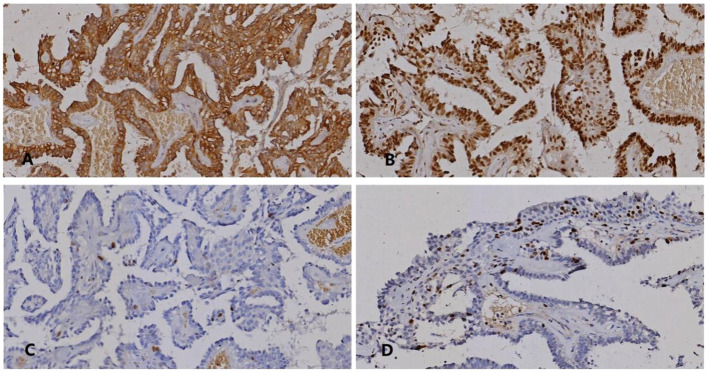
**(A)** The CK of tumor cells exhibits diffuse and strongly positive membranous expression (200×); **(B)** The TTF-1 of tumor cells demonstrates diffuse and strongly positive nuclear expression (200×); **(C)** The TG of tumor cells manifests as focal positive expression (200×); **(D)** The positive index of Ki−67 in tumor cells is relatively low (200×).

Pathological diagnosis: Based on the results of hematoxylin-eosin (HE) staining and immunohistochemical examination, a diagnosis of thyroid-like low-grade papillary adenocarcinoma is supported.

## Discussion

Nasopharyngeal tumors constitute a common type of neoplasm in the head and neck region. Nevertheless, TL-LGNPPA accounts for merely 0.38 to 0.48% of malignant nasopharyngeal tumors. In 1988, Wenig et al. (31). were the first to propose the concept of nasopharyngeal papillary adenocarcinoma (NPA). In 2005, Carrizo and Luna initially discovered that NPA expressed TTF-1 and named it thyroid-like low-grade papillary adenocarcinoma (1).

Upon literature retrieval and screening, a total of 58 cases, including the present case, were incorporated. Through the summary of **Table 1**, the age of onset of TL-LGNPPA has a wide range (9 to 71 years), with an average age of 38 years and a median age of 38.5 years. There is no significant difference in the incidence rate between males and females (1:1.14). The clinical symptoms of patients are mostly dominated by nasal obstruction, which may be accompanied by rhinorrhea, blood in the nasal discharge, epistaxis, a foreign body sensation in the nasopharynx, and other symptoms. A few may present symptoms such as dizziness and syncope, and it is also possible to be incidentally discovered during asymptomatic physical examinations. The majority of tumors originate from the roof of the nasopharynx (RON) and the posterior margin of the nasal septum (PMONP). The tumor size ranges from 0.3 to 4 cm. They are mostly grayish-red pedunculated polypoid masses with smooth edges and show invasive growth (**Table 1**). The age of onset in this case is relatively early and originates from the roof of the nasopharynx, which is consistent with previous literature reports.

Imaging examinations can also assist in its diagnosis. The study by Baba et al. proposed that CT examination reveals that the mass is located at the midline of the posterior margin of the nasal septum, adheres to the nasopharyngeal wall, does not invade adjacent structures, presents as a lobulated or smooth mass with clear edges, low internal density, and adjacent bone sclerosis, which can be considered for the imaging diagnosis of TL-LGNPPA (29). In this case, the mass is located on the roof of the nasopharynx. The enhanced scan shows significant heterogeneous enhancement of the lesion, slightly thickened nasopharyngeal mucosa, and significantly enhanced mucosa on enhanced scan. No destruction or absorption of the skull base bone is observed, which can suggest the diagnosis of TL-LGNPPA.

TL-LGNPPA typically exhibits a typical papillary and/or adenoid tubular configuration under microscopic examination, lined with single-layer cuboidal epithelial cells or single-layer columnar epithelial cells. In rare circumstances, it may also be lined with pseudostratified columnar epithelial cells. A tightly arranged fibrous vascular axis can be discerned in the interstitium. The nuclei are round to oval, mostly ground-glass-like, and nuclear grooves, pseudoinclusions, and psammoma bodies are readily observable. The cytoplasm is eosinophilic, with mild atypia and a scarcity of mitotic figures, often demonstrating an invasive growth pattern, and its structure is analogous to that of papillary thyroid carcinoma (PTC). Among the reported cases of TL-LGNPPA to date, there are 3 cases with spindle cell components (5, 8, 16, 30), 2 cases accompanied by squamous differentiation (12, 19), and 1 case with para-tumoral squamous cell carcinoma (22). In this case, no spindle cell components or squamous differentiation were identified, nor were other types of tumor architectures detected.

The immunohistochemical markers of TL-LGNPPA uniformly exhibit expressions of TTF-1 and CK, while cytokeratin 7 (CK7), cytokeratin 19 (CK19), and epithelial membrane antigen (EMA) typically present as strongly positive or diffusely positive expressions. TG is generally not expressed, but there have also been reports of focal and mild positive expression of TG (15, 17). The Ki-67 index is relatively low, and the range reported in the literature is from 1% to 60%, with approximately 98% of cases being <10% (**Table 1**). In this case, TTF-1 and CK show diffuse and strongly positive expressions, the Ki-67 index is approximately 5-10%, and TG shows focal positive expression, which is consistent with the previous literature reports.

TTF-1, a tissue-specific transcription factor with a homeodomain protein folding structure, is a member of the NKx2 family of tissue-specific transcription factors, and its normal expression is restricted to the lung and thyroid gland. The reason for the expression of TTF-1 in TL-LGNPPA remains unclear. Currently, the following hypotheses have been proposed: Firstly, TTF-1 may be involved in the development of the pharynx and larynx during the embryonic period or due to ectopic thyroid tissue (30). Secondly, the aberrant expression of TTF-1 might be attributed to the rearrangement of the NKX2–1 gene. Thirdly, the instability and reprogramming of cancer cell genes may lead to the differentiation and dysregulation of TTF-1/NKX2–1 (1, 16). However, none of these three mechanisms has supporting evidence and requires further investigation.

TG, an iodinated glycoprotein, is solely produced by mature thyroid tissue and stored within the follicular lumen, serving as the substrate for thyroid hormone synthesis. In TL-LGNPPA, TG is typically not expressed (2, 3). Nevertheless, given the focal positive expression of TG in this case, to rule out metastatic papillary thyroid carcinoma, the patient underwent ultrasound examination, which revealed a normal thyroid, and the thyroid function test also showed no abnormalities.

The diagnosis of TL-LGNPPA relies on the pathological morphology and the results of immunohistochemical staining, and the immunophenotype is conducive to its differential diagnosis. TL-LGNPPA, along with nasopharyngeal polymorphous low-grade adenocarcinoma (PLGA), metastatic papillary thyroid carcinoma (MPTC), and metastatic papillary adenocarcinoma of the lung, etc., are difficult to distinguish merely from the morphological perspective (5, 12). Positive nuclear staining of TTF-1 can be used to differentiate PLGA, but it is not applicable for differentiating MPTC and metastatic papillary adenocarcinoma of the lung. This requires additional immunohistochemical markers for aiding in the differential diagnosis: MPTC can concurrently express TG and paired box 8 (PAX-8), and metastatic papillary adenocarcinoma of the lung can express Naspin A. However, in TL-LGNPPA, TG, PAX-8, and Naspin A typically show negative expression (5).

The etiology and pathogenesis of TL-LGNPPA remain elusive at present. The occurrence and development of nasopharyngeal tumors are often closely associated with Epstein-Barr virus (EBV), but multiple literature reports and studies have indicated that the occurrence of TL-LGNPPA is not related to EBV (1, 12). Wang et al. conducted whole-exome sequencing on four cases and identified a total of 41 mutation sites. Among them, except for one case with a DEK driver gene mutation, no common genes or other classical driver gene mutations were detected, nor were microsatellite instability and HLA heterozygosity loss found. Additionally, copy number variations (deletions) in the 7q22.1-7q12 region were detected in two specimens, which encompassed genes such as RASA4, POLR2J2, SPDYE2, CCL3, and CCL4 (3). Wang et al. performed next-generation sequencing on four cases and detected a total of five mutant genes (FGFR4, PDK1, AXIN2, FOXL2, and PIK3C3), as well as copy number variations of MCL1 and STMN1. No repetitive genetic alterations were identified in the aforementioned studies, and the significance of the detected gene mutations remains unclear (9). Of course, the current number of detected cases is relatively small, presenting certain limitations, and whole-genome sequencing has not been conducted. Therefore, more cases and in-depth genomic studies are required.

The treatment of TL-LGNPPA primarily opts for surgical resection, and the surgical approach is selected based on the size and location of the tumor. In this case, the mass was resected through nasal endoscopic surgery, and the recovery was favorable. There was no recurrence or metastasis three months after the surgery. Due to the relatively short follow-up period, a long-term follow-up is necessary for further assessment. Among the cases with complete follow-up information reported in the current literature, there are no reports of postoperative recurrence or metastasis as of yet. Given the limited number of reported cases, the biological behavior of TL-LGNPPA still requires the accumulation of long-term follow-up data from more cases for further evaluation.

## Data Availability

The original contributions presented in the study are included in the article/supplementary material. Further inquiries can be directed to the corresponding authors.
